# Real-world evaluation of a transformer-based natural language processing system for identifying social determinants of health from routine clinical documentation

**DOI:** 10.1093/jamiaopen/ooag147

**Published:** 2026-07-31

**Authors:** Xiangren Wang, Jingchuan Guo, Yi Guo, Yao An Lee, Mattia Prosperi, Jiang Bian, Noah Hammarlund

**Affiliations:** Department of Health Services Research, Management and Policy, University of Florida, Gainesville, FL 32610, United States; Department of Pharmacy Practice, College of Pharmacy, Purdue University, West Lafayette, IN 47907, United States; Regenstrief Institute Inc, Indianapolis, IN 46202, United States; Department of Health Outcomes and Biomedical Informatics, University of Florida, Gainesville, FL 32610, United States; Department of Pharmaceutical Outcomes and Policy, University of Florida, Gainesville, FL 32611, United States; Department of Epidemiology, University of Florida, Gainesville, FL 32603, United States; Department of Biostatistics and Health Data Science, Indiana University, Indianapolis, IN 46202, United States; Department of Health Services Research, Management and Policy, University of Florida, Gainesville, FL 32610, United States

**Keywords:** social determinants of health, natural language processing, electronic health record

## Abstract

**Objectives:**

To evaluate the real-world performance of a transformer-based natural language processing (NLP) system for extracting Social Determinants of Health (SDoH) from clinical notes, using survey-based SDoH measures a reference comparators.

**Materials and Methods:**

This study was conducted at the University of Florida Health in adults with at least 2 clinical encounters in the prior year. A research survey was completed by 1001 participants; sampling targeted 50% Black patients to support subgroup analyses. Comparative analyses were restricted to the 414 participants who also had Epic SDoH survey data and clinical notes available for NLP extraction. We compared concepts extracted by the SOcial DeterminAnts (SODA) NLP pipeline against the independently administered research survey, which served as the primary reference standard, and against the structured Epic-embedded SDoH questionnaire. Nine domains were evaluated: abuse, alcohol use, drug use, education, financial constraints, housing, physical activity, social cohesion, and transportation. Sensitivity, specificity, positive predictive value, negative predictive value, and F1 scores were calculated by domain.

**Results:**

The NLP pipeline more consistently aligned with negative survey responses than with patient-reported social needs, although performance was lower in some domains, especially alcohol use and financial constraints. Sensitivity was higher only for alcohol use (55%); the lowest values were for abuse (5%), drug use (0%), and financial constraints (16%). These results cannot be attributed to SODA extraction alone. They reflect some combination of social information not being recorded in clinical notes, content that was recorded but not extracted, and mismatches between extracted concepts and survey definitions, and the present analysis cannot separate these contributions. The 2 surveys agreed only modestly with each other, so no single instrument provides a definitive ground truth.

**Conclusion:**

The pipeline more consistently aligned with negative survey responses than with patient-reported social needs. Because the 2 surveys agreed only modestly, the reference itself is imperfect, and apparent NLP performance depends in part on which survey is used as the comparator. Apparent gaps in NLP performance reflect both how social risks are recorded in clinical notes and how patients disclose them across different survey settings, in addition to limits of the extraction pipeline. Improving documentation practices, integrating locally tuned large language models, and monitoring subgroup performance may all be needed to make SDoH identification tools reliably detect social needs across patient populations.

## Background and significance

Social determinants of health (SDoH) are the non‑medical conditions of daily life, including the places where people live, work, learn, and age, that shape their health and their access to care.^1^^,^^2^ SDoH include factors such as housing stability, educational attainment, access to transportation, and employment status, which can substantially influence health outcomes and access to healthcare services.^3^ Health systems now prioritize identifying and addressing social needs to advance equity and meet care delivery goals.^4^ Evidence suggests that addressing social needs, such as housing insecurity, can reduce hospital admissions and emergency department visits.^5^

Despite the growing institutional focus, SDoH data remain under-captured in routine clinical workflows.^6^ Healthcare systems typically use 2 approaches. First, the ICD‑10 Z codes, which indicate socioeconomic and psychosocial circumstances, are available in structured electronic health record (EHR) data but are seldom used in practice.^4^^,^^6^^,^^7^ Second, structured EHR‑based surveys, which are delivered via patient portals or during clinical encounters, are used to capture broader domains but face barriers that limit consistent completion. Screening may be skipped under clinical time pressure, items and thresholds may vary across instruments, portal-based questionnaires may not be accessible to all patients, and patients may be reluctant to disclose sensitive needs within a clinical setting.^8–10^ Because of these limitations, providers often lack timely and accurate information about patients’ social risks.^11^ The gap is particularly consequential among high‑risk populations, where unmet social needs frequently contribute to preventable healthcare utilization and adverse clinical outcomes.^11^

An alternate source of SDoH data is unstructured documentation within EHR data. Patients may reveal social challenges during clinical encounters, which providers record in narrative notes. Advances in natural language processing (NLP) provide new ways to identify the existence of SDoH barriers in these free-text notes.^12^ In this study, we evaluated the SOcial DeterminAnts (SODA) NLP package, an open-source tool that uses fine-tuned transformer language models, notably GatorTron, to extract structured SDoH information from free-text clinical notes. We previously developed SODA and validated it on annotated corpora covering 19 categories of SDoH. SODA uses both concept extraction and relationship linking methods to capture detailed social context from narrative clinical documentation. Transformer models use attention to weigh how surrounding words shape meaning, capturing context beyond fixed keyword matching. We reported high benchmark performance, with strict F1 scores above 0.90, in controlled evaluations on expert-annotated note spans. The present study goes one step further by evaluating how well SODA-derived indicators align with patient-reported SDoH status captured by an independently administered, confidential research survey and the structured Epic-embedded questionnaire. The original SODA evaluation did not include patient-reported reference data.^12^ Although benchmark performance is high, SODA and similar NLP systems require real-world evaluation, where documentation is variable and patient populations are diverse. Comparable tools, including cTAKES, CLAMP, MetaMap, and medspaCy, also perform well on curated datasets but have rarely been tested in routine clinical settings where social information is inconsistently recorded.^13–17^

Although some NLP-based methods have been examined in real-world clinical settings, few have specifically used patient-reported data as the standard for evaluation.^12–17^ In this study, we evaluated the performance of a transformer-based NLP system applied to provider notes, using a confidential, independently administered research survey as the reference standard. We also compared NLP-derived SDoH data to responses from a structured SDoH survey embedded in a commonly adopted Epic EHR system. Additionally, we assessed agreement between the 2 survey modalities.

The significance of this study lies in its use of independently administered, confidential patient surveys as the reference standard, rather than structured EHR fields or annotated training data, offering a more realistic picture of what these tools can and cannot do. The findings have direct implications for how health systems decide to invest in informatics infrastructure, how documentation workflows are designed, and whether the tools deployed to identify social risk can be trusted to work consistently across different patient populations.

## Methods

### Study design and setting

We conducted a system-level evaluation of an NLP-based SDoH pipeline by comparing concepts extracted from retrospectively collected routine clinical notes using the SODA package with data from prospectively administered structured patient-reported surveys. All analyses were conducted using existing clinical documentation, and SODA was not used prospectively in patient care or clinical decision-making. Participants included adults who received care at UF Health between August 1, 2022, and August 1, 2023. UF Health is University of Florida’s academic health system, which operates 11 hospitals and more than 40 outpatient practices across North and Central Florida, with major campuses in Gainesville and Jacksonville.^18^^,^^19^ The health system delivers primary and specialty care through a broad network that spans both urban and rural communities.^18^^,^^19^

Eligible participants were 18 years or older and provided consent for data sharing at the time of their clinical encounters, identified from the UF Health EHR system (Epic, Verona, WI; version 2024). The participants’ inclusion criteria also required at least one clinical encounter at UF Health between August 2022 and August 2023 (the index date), and at least one encounter more than 365 days earlier.

A total of 1001 participants completed the research survey. Of these, 414 also had Epic SDoH survey data and clinical notes available for NLP extraction, and these 414 participants form the analytic sample for the NLP-versus-survey and survey-versus-survey comparisons reported below. The remaining 587 respondents lacked Epic SDoH survey data, clinical notes, or both, and were not included in the comparative analyses. The sampling frame was stratified to target 50% Black patients to ensure adequate power for subgroup analyses, given documented disparities in both SDoH burden and documentation. The analytic subset was 22.7% Black, well below the 50% target. This reflects substantial attrition between the source pool and the analytic sample: lower research survey response rates among Black patients, and differential availability of both Epic SDoH survey data and clinical notes within respondents, which disproportionately reduce Black representation. Additionally, we oversampled patients who previously completed an Epic EHR-based SDoH survey to facilitate comparisons across data sources. Participants were compensated $25 upon completing the research survey. The Institutional Review Board approved the study at the University of Florida (IRB202301129).

To better understand the sources of apparent false negatives (research survey = 1, SODA = 0), we conducted a targeted chart-note review of 60 false-negative cases, drawing a random sample of 10 cases from each of 6 priority domains with low sensitivity or high clinical relevance: abuse, drug use, financial constraints, housing, transportation, and physical activity. For each case, we examined 2 sources of evidence in parallel: (1) the SODA extraction record for the relevant domain (raw extracted text, mention, normalized value, and attributes) and (2) a simple keyword-based search of the linked clinical-note text for terms that would plausibly indicate the topic was discussed (eg, “drug use,” “marijuana,” or “substance abuse” for the drug-use domain). The keyword search is a transparent, lower-bound check on whether a topic is present in the note at all; it does not substitute for the SODA pipeline or for expert annotation. Each case was then assigned to 1 of 6 categories: not documented (topic absent from the note); documented but not extracted (topic present in the note but missed by SODA, or extracted positively but lost in patient-level aggregation); ambiguous or indirect documentation (weak or peripheral mention); documented but discordant (the EHR field captured the construct but the clinical answer differed from the research-survey answer, eg, “Drug use: No” in the structured social-history section while the patient reported drug use on the research survey); construct mismatch (the EHR field measures a different construct than the survey question, most clearly for physical activity); and unable to determine (the linked note was too brief or non-substantive, such as an attestation or short message). Classifications were AI-assisted using a structured decision rule combining the 2 evidence sources and were reviewed by the study team. This review was intended to characterize likely sources of discordance rather than serve as an independent expert annotation study and counts by domain and category are reported in Table S4.

### Data sources

We developed a structured patient survey encompassing 9 key SDoH domains derived from the World Health Organization’s SDoH framework. Domains were operationalized as binary indicators of disadvantage: abuse (intimate partner violence), alcohol use (any regular drinking), education (less than a college degree), financial constraints (difficulty affording basics), housing (unstable living situation), physical activity (lower than peers), social cohesion (low connectedness), and transportation (lack of reliable access).^20^ We used validated items from prior instruments.^21^ Most items were adapted from the 2 widely used screeners of PRAPARE (Protocol for Responding to and Assessing Patients’ Assets, Risks, and Experiences) and the Accountable Health Communities (AHC) tool.^22^ Additionally, validated instruments were incorporated to capture domains not fully addressed by those screeners, including the Exercise Vital Sign for physical activity,^23^ the Single Item Literacy Screener for health literacy,^24^ and the PHQ-2 for depression.^25^ Responses were dichotomized into binary indicators of disadvantage. Thresholds followed the definitions of the source instruments wherever possible. We used binary indicators because SODA produces binary, patient-level concept-presence outputs after aggregation, and the survey items were either binary or were collapsed to binary; item-by-item dichotomization rules for all 3 sources are provided in Table S5. Surveys were independently administered to 1001 eligible patients to ensure confidentiality and reduce potential biases associated with clinical contexts.

### Natural language processing approach

We used the SODA NLP pipeline developed at UF Health to extract structured SDoH indicators from clinical notes in the same cohort of survey participants. In the remainder of this paper, references to the NLP system or to NLP-derived indicators refer specifically to SODA, whereas NLP alone denotes the broader class of NLP methods. SODA is an open-source toolkit built on transformer-based language models, notably GatorTron.^12^ We aggregated SODA outputs into binary domain indicators. For each patient, we processed all available clinical notes from the study window (August 1, 2022, through August 1, 2023). This included inpatient and outpatient encounters and notes written by physicians, nurses, advanced practice providers, and social workers; portal messages and addenda were included when available. SODA outputs were aggregated to the patient level by mapping extracted concepts and their attributes to the 9 SDoH domains used in this study. A domain was classified as present (1) if any corresponding concept appeared in any of the patient’s notes during the study window, and absent (0) otherwise. Negation and other contextual modifiers (eg, historical, family-history) were handled within the SODA pipeline and were not counted as present. Table S5 lists the full mapping from SODA concept categories to the 9 binary domains. This patient-level rule allowed direct comparison with the binary survey indicators but is likely to misclassify cases when relevant content is recorded indirectly or in language that does not map cleanly onto the survey definition.

### Structured EHR-based survey

For the same cohort of survey participants, data from an Epic-integrated SDoH survey were retrieved for patients who had previously completed assessments through routine clinical encounters. The Epic EHR survey includes structured items routinely administered via the MyChart patient portal or during in-clinic intake workflows. The Epic instrument did not include a drug-use item. Therefore, the drug-use domain was not available from the EHR survey. Responses were summarized into binary domain-specific indicators to make them comparable to the NLP and research survey data. Table S5 lists, by domain, the survey items used, and the rule applied to dichotomize each item into a binary indicator of disadvantage. We used the same domain definitions for the research survey, the Epic-embedded survey, and the SODA-derived indicators.

### Statistical analysis

We calculated standard classification performance metrics, including sensitivity, specificity, positive predictive value (PPV), negative predictive value (NPV), false positives and negatives, and F1 scores, for each of the 9 SDoH domains. The research survey served as the primary reference standard for evaluating SODA: it was designed for this study and completed confidentially, outside any clinical encounter. For the survey-to-survey comparison, we treat the research survey as a reference comparator rather than a gold standard, since both instruments depend on what patients chose to disclose in different settings. The metrics in Table 4 should, therefore, be read as agreement between 2 patient-reported sources, not as classification accuracy. Three comparisons were performed: (1) NLP performance against the research survey, (2) NLP performance against Epic EHR survey, and (3) survey-to-survey agreement. All analyses focused on capturing whether respondents reported experiencing barriers within each domain, without considering the frequency or timing of reported barriers. Statistical analyses were performed using R software (v4.4.2, R Core Team 2024).

**Table 1.** Demographic characteristics of the study population, *n* = 414.

	Mean
Age (years)	55
Sex	
Male	0.290
Race/Ethnicity	
Black	0.227

Demographic and clinical characteristics of the study population. Participant characteristics reflect the analytic sample (*n* = 414), a subset of 1001 total survey respondents with available Epic SDoH survey data and clinical notes. Values represent the mean for continuous variables and proportions for categorical variables.

**Table 2.** NLP performance against research survey.

Domain	*N*	Research survey positives	NLP positives	Accuracy	Sensitivity	Specificity	PPV	NPV	False POS	False NEG	F1
Abuse	414	18	49	84.3	5.6	87.9	2	95.3	12.1	94.4	3
Alcohol	414	60	199	53.4	55	53.1	16.6	87.4	46.9	45	25.5
Education	414	87	34	75.1	10.3	92.4	26.5	79.5	7.6	89.7	14.9
Financial constraints	414	177	58	57.2	16.4	87.8	50	58.4	12.2	83.6	24.7
Drug	414	15	44	85.7	0	89	0	95.9	11	100	0
Housing	414	22	0	94.7	0	100	0	94.7	0	100	0
Physical	414	76	14	80.2	5.3	97	28.6	82	3	94.7	8.9
Social cohesion	414	63	16	82.9	6.3	96.6	25	85.2	3.4	93.7	10.1
Transportation	414	53	0	87.2	0	100	0	87.2	0	100	0
Average score				77.86	10.99	89.31	16.52	85.07	10.69	89.01	9.68

Domain-level performance of the NLP system against the research survey reference. Metrics are expressed as percentages and include overall accuracy, sensitivity, specificity, PPV, NPV, and F1 score.

**Table 3.** NLP performance against EHR survey.

Domain	*N*	EHR survey positives	NLP positives	Accuracy	Sensitivity	Specificity	PPV	NPV	False POS	False NEG	F1
Abuse	414	34	49	84.3	26.5	89.5	18.4	93.2	10.5	73.5	21.7
Alcohol	414	65	199	46.4	32.3	49	10.6	79.5	51	67.7	15.9
Education	414	269	34	37.9	8.6	92.4	67.6	35.3	7.6	91.4	15.2
Financial constraints	414	249	58	42.3	13.7	85.5	58.6	39.6	14.5	86.3	22.1
Housing	414	29	0	93	0	100	0	93	0	100	0
Physical	414	107	14	72.2	2.8	96.4	21.4	74	3.6	97.2	5
Social cohesion	414	73	16	80.4	5.5	96.5	25	82.7	3.5	94.5	9
Transportation	414	26	0	93.7	0	100	0	93.7	0	100	0
Average score				68.78	11.18	88.66	25.20	73.88	11.34	88.83	11.11

Domain-level performance of the NLP system against the Epic EHR-embedded survey reference. Metrics are expressed as percentages and include sensitivity, specificity, PPV, NPV, and F1 score. The Epic EHR survey does not include a drug-use item; the drug-use domain is omitted from the table. Results again show modest-to-low sensitivity and relatively high specificity across domains, indicating under-detection of SDoH concepts documented in structured EHR surveys.

**Table 4.** Survey-to-survey agreement.

Domain	*N*	Research survey positives	EHR survey positives	Accuracy	Sensitivity	Specificity	PPV	NPV	False POS	False NEG	F1
Abuse	414	18	34	87.9	5.6	91.7	2.9	95.5	8.3	94.4	3.8
Alcohol	414	60	65	75.1	18.3	84.7	16.9	86	15.3	81.7	17.6
Education	414	87	269	42	66.7	35.5	21.6	80	64.5	33.3	32.6
Financial constraints	414	177	249	45.4	56.5	37.1	40.2	53.3	62.9	43.5	46.9
Housing	414	22	29	89.1	13.6	93.4	10.3	95.1	6.6	86.4	11.8
Physical	414	76	107	65	25	74	17.8	81.4	26	75	20.8
Social cohesion	414	63	73	72.5	17.5	82.3	15.1	84.8	17.7	82.5	16.2
Transportation	414	53	26	82.4	5.7	93.6	11.5	87.1	6.4	94.3	7.6
Average score				69.93	26.11	74.04	17.04	82.90	25.96	73.89	19.66

Domain-level agreement between the research survey and the structured Epic EHR survey. Metrics are expressed as percentages and include sensitivity, specificity, PPV, NPV, and F1 score. Results show variable alignment between survey modalities, with generally moderate sensitivity and specificity, suggesting that survey context and administration method influence patient disclosure of SDoH factors. Because neither survey represents a definitive measure of true SDoH status, these metrics should be interpreted as directional agreement measures rather than diagnostic performance.

To assess whether NLP performance varied across patient subgroups, we stratified analyses by race (Black vs non-Black), sex, and age (≥65 vs <65). For each subgroup, we compared the proportion of cases where NLP and survey results agreed versus disagreed across subgroup levels. Chi-square tests were used to evaluate stratified differences in overall agreement rates.

### Patient involvement

Patients and members of the public were not involved in the design, conduct, or dissemination of this research. However, patient participation was central to the study, as independently administered patient-reported surveys served as the primary reference standard for evaluating SDoH extracted from clinical documentation.

## Results

### Participant characteristics

Of the 1001 survey respondents, 414 also had Epic SDoH survey data and clinical notes suitable for NLP analysis. This subset constituted the analytic sample for all comparative analyses. Table 1 presents the characteristics of these 414 participants: 29.5% were aged ≥65 years and 29.0% were male. By race and ethnicity, 22.7% were Black, 4.3% were Hispanic, and Asian representation was negligible (0.2%).

### NLP performance against research survey

Table 2 presents the results of the comparison between the NLP-derived and the research survey-derived indicators. With the research survey as the reference standard, we identified that NLP-derived SDoH indicators exhibited consistently low sensitivity across all evaluated domains, ranging from 0% (drug use, housing, and transportation) to 16.4% (financial constraints). In contrast, specificity was greater than 85% across most domains, indicating that SODA-derived positive indicators were uncommon among patients who did not report barriers on the research survey.

Positive predictive values were low (generally below 30%), suggesting NLP-identified barriers rarely corresponded to actual patient-reported barriers. Negative predictive values were also high in most domains, but this should be interpreted alongside the low prevalence of several SDoH barriers, and the low sensitivity observed across domains. Performance was particularly low in several domains, including financial constraints (sensitivity: 16%, specificity: 88%), drug use, and physical activity (all with sensitivity near zero).

In the stratified analyses, agreement rates between NLP and the research survey were broadly similar across race, sex, and age groups. Out of all domain and subgroup comparisons, only 2 reached statistical significance: Physical activity by Black race (*P* = .014) and social cohesion by age ≥65 (*P* = .024). For the physical activity domain, performance was uniformly low across groups but especially weak among Black patients, where SODA did not identify any true positive cases. For social cohesion, sensitivity was low across both age groups, but older adults showed higher specificity and overall accuracy. Analyses for Asian and Hispanic patients were limited by sparse and inconsistent race/ethnicity data, whereas Black race was recorded consistently enough to support powered Black vs non-Black comparisons. Full domain-level stratified performance metrics by race, sex, and age are provided in Table S1 (research survey reference, corresponding to Table 2), Table S2 (Epic-embedded survey reference, corresponding to Table 3), and Table S3 (survey-to-survey comparison, corresponding to Table 4).

### NLP performance against Epic EHR survey

Table 3 presents the results of the comparison between the NLP-derived and the Epic survey-derived indicators. Comparisons of NLP performance against the Epic EHR-based survey yielded similar patterns of limited sensitivity (generally <30%) and consistently high specificity (>85%). The drug-use domain was excluded from this comparison because it is not collected by the Epic survey. PPVs remained low, with only marginal improvements compared to the research survey-based results. NPVs were higher than PPVs in most domains, but, as with the research survey comparison, should be interpreted alongside low prevalence and low sensitivity. F1 scores were low across domains (<23%), particularly in domains such as alcohol use and physical activity, where NLP demonstrated negligible improvement when benchmarked against the structured EHR survey.

### Survey-to-survey agreement

Table 4 presents the results of the comparison between the Epic survey-derived and the research survey-derived indicators. Agreement between the independently administered research survey and the Epic EHR survey was low across most domains. Using the research survey as the reference comparator, directional sensitivity was particularly low for abuse (5.6%) and transportation (5.7%). Other directional agreement metrics also varied substantially across domains, with low positive predictive values and F1 scores. These findings indicate substantial discordance between clinic-embedded assessments and independently administered research surveys, and they underscore that neither survey should be interpreted as a definitive measure of true SDoH status.

## Discussion

This study evaluates the real-world performance of a transformer-based NLP system (SODA) for SDoH extraction from routine clinical documentation, using independent patient-reported survey measures as comparators and the research survey as the primary reference source. By comparing SDoH signals extracted from routine clinical notes with an independently administered patient survey and structured Epic-embedded questionnaires, we observed substantial gaps between patient reported social needs and what is captured through routine documentation, whether via NLP derived concepts or structured EHR questionnaires.

While previous research demonstrated that NLP methods, including transformer-based models, achieved high accuracy and F1 scores (>0.90) on benchmark datasets for SDoH extraction,^12–16^ our study reveals limitations when applied to routine clinical documentation. Sensitivity ranged from 0% to 55% overall but was ≤16% for 8 of 9 domains, excluding alcohol use. This highlights a structural gap between patient-reported social risks collected through research surveys and the social information routinely recorded in clinical documentation that serves as input to the SODA pipeline. Low sensitivity in this study likely reflects 2 distinct problems. In some domains, social information was absent from clinical notes entirely. In others, relevant information was present but not reliably extracted by the NLP pipeline. To distinguish among possible explanations, we conducted a targeted chart-note error analysis on 60 false-negative cases (10 per priority domain across abuse, drug use, financial constraints, housing, transportation, and physical activity) and classified each into 1 of 6 categories using the SODA extraction record and the linked clinical note (Table S4). In the reviewed cases, 4 of 60 (7%) were clearly attributable to extraction failure, where the topic was visible in the note, but SODA did not capture it. The larger share, 21 of 60 (35%), reflected discordance between the patient’s clinical answer and the research-survey answer (eg, “Drug use: No” recorded in the social-history section while the patient reported drug use on the confidential research survey), construct mismatch between the EHR field and the survey question (most clearly in physical activity, where the EHR captures absolute days/week of exercise while the survey asked for relative activity compared with peers), or linked notes that were too brief to adjudicate (eg, attestations and short messages). A smaller number of reviewed cases were either not documented in the note or only indirectly referenced. The targeted nature of this review, and the fact that it relied on a structured first-pass classification rather than independent expert annotation, mean that the counts in Table S4 should be read as descriptive rather than as precise estimates of the contribution of each source to overall sensitivity. The pattern, however, is informative: extraction failure was not the dominant pattern in this sample, and apparent under-detection by SODA appeared to reflect a combination of how social information was recorded, how patients disclosed it across different contexts, and how EHR fields were defined relative to the survey. As a transparent baseline check, the simple keyword search used in this review was unable to recover most of the patient-reported barriers either, indicating that the apparent gap between research-survey responses and what is recoverable from these notes is not specific to the transformer-based SODA pipeline. A formal head-to-head benchmark against concept-recognition tools such as cTAKES, CLAMP, or MetaMap on the same notes remains a useful next step.

Although specificity and NPV were relatively high in most domains, these metrics should not be interpreted as sufficient evidence that SODA can rule out social needs in clinical practice. Given the low sensitivity, SODA-derived indicators are better understood as incomplete signals from routine documentation rather than comprehensive measures of patient social risk. The key finding is that note-derived indicators aligned more consistently with negative survey responses than with patient-reported barriers. Together, these findings highlight a gap between benchmark performance and real-world use. Improving SDoH extraction will likely require stronger documentation practices and careful integration of NLP-derived indicators into clinical and population health workflows.

The performance gaps we observed likely reflect wide variation in documentation practices and inconsistencies in clinician engagement with SDoH topics during clinical encounters. Patients’ comfort with disclosing sensitive or stigmatizing needs also varies, which further limits what gets recorded.^26^ The NLP-based approach showed more limited capture when identification relied on subtle or contextual interpretation, particularly for sensitive domains, such as financial hardship, substance use, and experiences of abuse. Financial constraints were the most prevalent domain in our sample. However, sensitivity in this domain was among the lowest, suggesting that patients with the greatest social burden may be least reliably identified. These patterns are consistent with prior research indicating lower disclosure rates and less explicit documentation of stigmatized or sensitive conditions in routine clinical records.^27^ Clinicians often use indirect language or omit information, and current NLP systems have difficulty parsing these nuances, highlighting the complexity of using unstructured clinical notes as sources of social risk data.^28^ The alcohol domain show a different pattern. Notes mention drinking in many forms, including social, occasional, and problematic, while the research survey targeted a narrower notion of risky drinking, so SODA classified more patients as positive than the survey did. These findings suggest that discordance may arise partly from mismatched domain definitions, not only from documentation gaps or extraction failure. Aligning extracted concepts more closely with survey thresholds may improve apparent performance and provide a more clinically meaningful evaluation.

Additionally, agreement between the independently administered research survey and the structured Epic EHR-embedded survey was low across most domains. This degree of discordance is not unique to our study. It reflects the broader reality of SDoH measurement, where no standardized screening instrument exists, even area- and individual-level social-risk measures align poorly, and routine SDoH screening in clinical practice remains inconsistent and incomplete.^8^^,^^9^^,^^10^^,^^29^ Patient-reported outcomes may vary by survey modality and context.^26^^,^^30^ Confidential, independently administered surveys may elicit more disclosure of sensitive risks than surveys completed during clinical encounters, where patients may have stronger privacy concerns.^26^^,^^30^ The small financial incentive in our study may also have increased completion or disclosure. This is consistent with prior evidence that disclosure of sensitive behaviors varies systematically with how patient-reported outcomes are collected. Clinic-based EHR surveys may, therefore, understate social risk and miss patients who would benefit from intervention. The low agreement between the 2 surveys also limits any simple interpretation of NLP “accuracy” in this study. Rather than treating any single instrument as a perfect gold standard for a patient’s true SDoH status, we view this analysis as an evaluation of how note-derived SDoH indicators align with patient-reported SDoH measures collected under different conditions. Apparent NLP performance in our results, therefore, depends in part on which patient-reported source is used as the comparator.

Our findings carry important implications for clinical informatics and health policy. First, relying solely on NLP-derived note extraction or on structured EHR-based surveys may not be sufficient for comprehensive SDoH screening, given the sensitivity gaps we observed. A multimodal approach to integrating multiple data sources, including patient-reported outcomes collected via confidential, independently administered surveys, may be necessary to capture the true extent of social risks. Clinicians and healthcare organizations should consider how survey context, workflow design, and informatics tools can be used to encourage disclosure and support more accurate reporting.

Second, continued methodological enhancements of NLP-based systems are essential. Advances in LLM technology may offer new opportunities to enhance SDoH extraction from clinical documentation, but further research is needed to determine how to best use these tools for real-world use. At the same time, clearer standards and guidance for how and why clinicians document social needs could substantially improve the quality and consistency of source data to improve the utility of both current and future NLP approaches. Strengthening documentation practices alongside technical innovation will enable more effective identification and intervention for patients facing social risks.

Third, fair performance across different groups remains a key concern when developing informatics tools for SDoH. While we designed our sampling to support subgroup analyses, the stratification results showed that NLP under-detection was broadly consistent across demographic groups. Only 2 isolated differences emerged: for physical activity among Black patients, NLP identified zero true positives, and for social cohesion among older adults, specificity was higher compared with younger adults. These results suggest that performance deficits are broad rather than group-specific, underscoring the need for continued work to ensure equitable accuracy across populations.

### Limitations and future directions

Our study has several limitations. While the single health system design at UF Health provides a detailed look at local documentation practices, it restricts generalizability. Other race and ethnicity strata, including Hispanic and Asian, were too sparsely and inconsistently recorded in the EHR to support meaningful analysis. Future work should ensure broader representation across patient groups. Additionally, lower research survey response rates among Black patients and differential availability of Epic survey data resulted in an analytic sample that was 22.7% Black, well below the 50% target. This represents substantial attrition relative to the original sampling design, may have introduced selection bias into the subgroup analyses, and should be considered when interpreting Black vs non-Black comparisons.

Using binary indicators for SDoH outcomes prevented us from assessing how severe barriers were or how they changed over time. Longitudinal analyses with graded SDoH measures would yield deeper insights into the clinical and operational value of both NLP and structured surveys. Although the SODA NLP pipeline was trained on a large set of clinical notes, it still struggled on new or more diverse patient groups, likely due to domain shifts in documentation style, vocabulary, patient mix, and the prevalence of specific SDoH concepts, leading to misclassification and reduced sensitivity. Emerging LLM-based approaches may offer better adaptability and generalizability, and future research should test that promise and how best to implement them in practice. Two further limitations are worth noting. The chart-note error analysis we conducted (Table S4) was a structured first-pass review combining the SODA extraction record with a search of the linked clinical note. The analysis also surfaced an unexpected finding worth flagging: for 20 of the 60 cases reviewed (33%), the linked clinical note returned by our note-extraction pipeline was a brief stub (attestation, phone message) too short to assess for SDoH content, even though SODA was applied to all available notes for each patient during the analysis. This means the linked-note slice that supports a portion of our error analysis is not fully representative of the underlying clinical record, and a more comprehensive note retrieval would be needed to characterize the “Unable to determine” cases. Finally, the analytic sample of 414 patients includes few true positives in low-prevalence domains such as housing, drug use, and abuse, so domain-specific estimates carry wide uncertainty.

## Conclusion

This study represents a systematic evaluation of transformer-based NLP for SDoH using independent patient-reported outcomes as the reference standard, rather than annotated corpora or structured EHR fields, within a large academic health system. Our study demonstrates that, when applied to routine clinical documentation, current informatics-driven approaches to capturing SDoH face important structural limitations. NLP extraction from clinical narratives more consistently aligned with negative survey responses than with patient-reported barriers, but the low sensitivity limits its use for identifying patients with social needs. The apparent gap reflects some combination of incomplete documentation, limits of context-dependent extraction by SODA, and mismatches between extracted concepts and survey items, and the present analysis cannot separate these contributions. Gaps were largest in domains such as drug use and physical activity, where social information is often described indirectly or omitted from notes. Addressing these gaps will require both ongoing methodological improvements in NLP and more effective documentation practices within clinical settings. Accurately identifying social needs is a prerequisite for effective intervention, and these findings underscore the need for cautious interpretation of SDoH signals derived from routine clinical data.

Going forward, some of the gaps identified in this study may be addressed through adaptive models, including large language models tailored to EHR documentation, as well as emerging AI-assisted clinical documentation tools (eg, ambient documentation systems such as Epic Ambient). However, the extent to which these approaches improve the capture of accurate and reliable SDoH information in routine clinical notes remains uncertain and warrants careful evaluation. The combination of information from clinical notes and structured SDoH screenings, with discrepancies prompting follow-up, may offer a more robust approach to characterizing social risk in practice. In parallel, clinicians may benefit from concise documentation templates, smart phrases, and trauma-informed training to support discussion and recording of social needs. Finally, performance should be monitored over time, across settings, and across patient subgroups as these tools evolve and are adopted.

## Supplementary Material

ooag147_Supplementary_Data

## Data Availability

The data underlying this study are not publicly available due to patient privacy, institutional policy, and data use restrictions under the approved University of Florida IRB protocol (IRB202301129). Clinical data were obtained from the University of Florida Health Integrated Data Repository (IDR) under institutional data use agreements. The research survey data contain sensitive, identifiable health information. R scripts used to calculate performance metrics are available from the corresponding author upon reasonable request.
