# Supplementary material for: Real-world evaluation of a transformer-based natural language processing system for identifying social determinants of health from routine clinical documentation
Source: JAMIA Open. 2026 Jul 31;9(4):ooag147. doi: 10.1093/jamiaopen/ooag147 (PMC13427761; doi:10.1093/jamiaopen/ooag147)
Supplement: ooag147_Supplementary_Data [file ooag147_supplementary_data.docx]

**Supplementary Materials**

**Supplementary Table S1. Stratified performance of SODA-derived NLP indicators against the research survey**

*Reference: Research Survey. Evaluated: NLP-derived (SODA from clinical notes). Domain-level performance metrics by Age (≥ 65 vs. < 65), Race (Black vs. Non-Black), and Sex (Female vs. Male). Metrics are expressed as percentages and include sensitivity, specificity, positive predictive value (PPV), negative predictive value (NPV), false-positive rate, false-negative rate, and F1 score. The full-cohort cells reproduce paper Table 2.*

**Age ≥ 65 (n = 122)**

| **Domain** | **N** | **Ref +** | **Pred +** | **Accuracy** | **Sensitivity** | **Specificity** | **PPV** | **NPV** | **False POS** | **False NEG** | **F1** |
| --- | --- | --- | --- | --- | --- | --- | --- | --- | --- | --- | --- |
| **Abuse** | 122 | 3 | 15 | 85.2 | 0 | 87.4 | 0 | 97.2 | 12.6 | 100 | 0 |
| **Alcohol** | 122 | 19 | 56 | 53.3 | 47.4 | 54.4 | 16.1 | 84.8 | 45.6 | 52.6 | 24 |
| **Education** | 122 | 25 | 7 | 78.7 | 12 | 95.9 | 42.9 | 80.9 | 4.1 | 88 | 18.8 |
| **Financial Constraints** | 122 | 39 | 20 | 64.8 | 20.5 | 85.5 | 40 | 69.6 | 14.5 | 79.5 | 27.1 |
| **Drug** | 122 | 3 | 10 | 89.3 | 0 | 91.6 | 0 | 97.3 | 8.4 | 100 | 0 |
| **Housing** | 122 | 3 | 0 | 97.5 | 0 | 100 | 0 | 97.5 | 0 | 100 | 0 |
| **Physical** | 122 | 14 | 3 | 86.1 | 0 | 97.2 | 0 | 88.2 | 2.8 | 100 | 0 |
| **Social Cohesion** | 122 | 13 | 4 | 87.7 | 7.7 | 97.2 | 25 | 89.8 | 2.8 | 92.3 | 11.8 |
| **Transportation** | 122 | 13 | 0 | 89.3 | 0 | 100 | 0 | 89.3 | 0 | 100 | 0 |

**Age < 65 (n = 292)**

| **Domain** | **N** | **Ref +** | **Pred +** | **Accuracy** | **Sensitivity** | **Specificity** | **PPV** | **NPV** | **False POS** | **False NEG** | **F1** |
| --- | --- | --- | --- | --- | --- | --- | --- | --- | --- | --- | --- |
| **Abuse** | 292 | 15 | 34 | 83.9 | 6.7 | 88.1 | 2.9 | 94.6 | 11.9 | 93.3 | 4.1 |
| **Alcohol** | 292 | 41 | 143 | 53.4 | 58.5 | 52.6 | 16.8 | 88.6 | 47.4 | 41.5 | 26.1 |
| **Education** | 292 | 62 | 27 | 73.6 | 9.7 | 90.9 | 22.2 | 78.9 | 9.1 | 90.3 | 13.5 |
| **Financial Constraints** | 292 | 138 | 38 | 54.1 | 15.2 | 89 | 55.3 | 53.9 | 11 | 84.8 | 23.9 |
| **Drug** | 292 | 12 | 34 | 84.2 | 0 | 87.9 | 0 | 95.3 | 12.1 | 100 | 0 |
| **Housing** | 292 | 19 | 0 | 93.5 | 0 | 100 | 0 | 93.5 | 0 | 100 | 0 |
| **Physical** | 292 | 62 | 11 | 77.7 | 6.5 | 97 | 36.4 | 79.4 | 3 | 93.5 | 11 |
| **Social Cohesion** | 292 | 50 | 12 | 80.8 | 6 | 96.3 | 25 | 83.2 | 3.7 | 94 | 9.7 |
| **Transportation** | 292 | 40 | 0 | 86.3 | 0 | 100 | 0 | 86.3 | 0 | 100 | 0 |

**Black (n = 94)**

| **Domain** | **N** | **Ref +** | **Pred +** | **Accuracy** | **Sensitivity** | **Specificity** | **PPV** | **NPV** | **False POS** | **False NEG** | **F1** |
| --- | --- | --- | --- | --- | --- | --- | --- | --- | --- | --- | --- |
| **Abuse** | 94 | 5 | 5 | 89.4 | 0 | 94.4 | 0 | 94.4 | 5.6 | 100 | 0 |
| **Alcohol** | 94 | 8 | 48 | 55.3 | 87.5 | 52.3 | 14.6 | 97.8 | 47.7 | 12.5 | 25 |
| **Education** | 94 | 28 | 9 | 67 | 10.7 | 90.9 | 33.3 | 70.6 | 9.1 | 89.3 | 16.2 |
| **Financial Constraints** | 94 | 55 | 13 | 44.7 | 14.5 | 87.2 | 61.5 | 42 | 12.8 | 85.5 | 23.5 |
| **Drug** | 94 | 2 | 12 | 85.1 | 0 | 87 | 0 | 97.6 | 13 | 100 | 0 |
| **Housing** | 94 | 10 | 0 | 89.4 | 0 | 100 | 0 | 89.4 | 0 | 100 | 0 |
| **Physical** | 94 | 20 | 2 | 76.6 | 0 | 97.3 | 0 | 78.3 | 2.7 | 100 | 0 |
| **Social Cohesion** | 94 | 19 | 5 | 76.6 | 5.3 | 94.7 | 20 | 79.8 | 5.3 | 94.7 | 8.3 |
| **Transportation** | 94 | 20 | 0 | 78.7 | 0 | 100 | 0 | 78.7 | 0 | 100 | 0 |

**Non-Black (n = 320)**

| **Domain** | **N** | **Ref +** | **Pred +** | **Accuracy** | **Sensitivity** | **Specificity** | **PPV** | **NPV** | **False POS** | **False NEG** | **F1** |
| --- | --- | --- | --- | --- | --- | --- | --- | --- | --- | --- | --- |
| **Abuse** | 320 | 13 | 44 | 82.8 | 7.7 | 86 | 2.3 | 95.7 | 14 | 92.3 | 3.5 |
| **Alcohol** | 320 | 52 | 151 | 52.8 | 50 | 53.4 | 17.2 | 84.6 | 46.6 | 50 | 25.6 |
| **Education** | 320 | 59 | 25 | 77.5 | 10.2 | 92.7 | 24 | 82 | 7.3 | 89.8 | 14.3 |
| **Financial Constraints** | 320 | 122 | 45 | 60.9 | 17.2 | 87.9 | 46.7 | 63.3 | 12.1 | 82.8 | 25.1 |
| **Drug** | 320 | 13 | 32 | 85.9 | 0 | 89.6 | 0 | 95.5 | 10.4 | 100 | 0 |
| **Housing** | 320 | 12 | 0 | 96.2 | 0 | 100 | 0 | 96.2 | 0 | 100 | 0 |
| **Physical** | 320 | 56 | 12 | 81.2 | 7.1 | 97 | 33.3 | 83.1 | 3 | 92.9 | 11.8 |
| **Social Cohesion** | 320 | 44 | 11 | 84.7 | 6.8 | 97.1 | 27.3 | 86.7 | 2.9 | 93.2 | 10.9 |
| **Transportation** | 320 | 33 | 0 | 89.7 | 0 | 100 | 0 | 89.7 | 0 | 100 | 0 |

**Female (n = 294)**

| **Domain** | **N** | **Ref +** | **Pred +** | **Accuracy** | **Sensitivity** | **Specificity** | **PPV** | **NPV** | **False POS** | **False NEG** | **F1** |
| --- | --- | --- | --- | --- | --- | --- | --- | --- | --- | --- | --- |
| **Abuse** | 294 | 12 | 30 | 85.7 | 0 | 89.4 | 0 | 95.5 | 10.6 | 100 | 0 |
| **Alcohol** | 294 | 40 | 136 | 55.1 | 55 | 55.1 | 16.2 | 88.6 | 44.9 | 45 | 25 |
| **Education** | 294 | 63 | 25 | 74.1 | 9.5 | 91.8 | 24 | 78.8 | 8.2 | 90.5 | 13.6 |
| **Financial Constraints** | 294 | 130 | 40 | 56.5 | 16.2 | 88.4 | 52.5 | 57.1 | 11.6 | 83.8 | 24.7 |
| **Drug** | 294 | 12 | 31 | 85.4 | 0 | 89 | 0 | 95.4 | 11 | 100 | 0 |
| **Housing** | 294 | 15 | 0 | 94.9 | 0 | 100 | 0 | 94.9 | 0 | 100 | 0 |
| **Physical** | 294 | 59 | 11 | 78.9 | 6.8 | 97 | 36.4 | 80.6 | 3 | 93.2 | 11.4 |
| **Social Cohesion** | 294 | 43 | 14 | 83.3 | 9.3 | 96 | 28.6 | 86.1 | 4 | 90.7 | 14 |
| **Transportation** | 294 | 37 | 0 | 87.4 | 0 | 100 | 0 | 87.4 | 0 | 100 | 0 |

**Male (n = 120)**

| **Domain** | **N** | **Ref +** | **Pred +** | **Accuracy** | **Sensitivity** | **Specificity** | **PPV** | **NPV** | **False POS** | **False NEG** | **F1** |
| --- | --- | --- | --- | --- | --- | --- | --- | --- | --- | --- | --- |
| **Abuse** | 120 | 6 | 19 | 80.8 | 16.7 | 84.2 | 5.3 | 95 | 15.8 | 83.3 | 8 |
| **Alcohol** | 120 | 20 | 63 | 49.2 | 55 | 48 | 17.5 | 84.2 | 52 | 45 | 26.5 |
| **Education** | 120 | 24 | 9 | 77.5 | 12.5 | 93.8 | 33.3 | 81.1 | 6.2 | 87.5 | 18.2 |
| **Financial Constraints** | 120 | 47 | 18 | 59.2 | 17 | 86.3 | 44.4 | 61.8 | 13.7 | 83 | 24.6 |
| **Drug** | 120 | 3 | 13 | 86.7 | 0 | 88.9 | 0 | 97.2 | 11.1 | 100 | 0 |
| **Housing** | 120 | 7 | 0 | 94.2 | 0 | 100 | 0 | 94.2 | 0 | 100 | 0 |
| **Physical** | 120 | 17 | 3 | 83.3 | 0 | 97.1 | 0 | 85.5 | 2.9 | 100 | 0 |
| **Social Cohesion** | 120 | 20 | 2 | 81.7 | 0 | 98 | 0 | 83.1 | 2 | 100 | 0 |
| **Transportation** | 120 | 16 | 0 | 86.7 | 0 | 100 | 0 | 86.7 | 0 | 100 | 0 |

**Supplementary Table S2. Stratified performance of SODA-derived NLP indicators against the Epic-embedded EHR survey**

*Reference: Epic-embedded EHR Survey. Evaluated: NLP-derived (SODA from clinical notes). Stratified by Age, Race, and Sex. The Drug domain is omitted because the Epic survey did not include a drug-use item. The full-cohort cells reproduce paper Table 3.*

**Age ≥ 65 (n = 122)**

| **Domain** | **N** | **Ref +** | **Pred +** | **Accuracy** | **Sensitivity** | **Specificity** | **PPV** | **NPV** | **False POS** | **False NEG** | **F1** |
| --- | --- | --- | --- | --- | --- | --- | --- | --- | --- | --- | --- |
| **Abuse** | 122 | 7 | 15 | 85.2 | 28.6 | 88.7 | 13.3 | 95.3 | 11.3 | 71.4 | 18.2 |
| **Alcohol** | 122 | 14 | 56 | 49.2 | 28.6 | 51.9 | 7.1 | 84.8 | 48.1 | 71.4 | 11.4 |
| **Education** | 122 | 70 | 7 | 43.4 | 5.7 | 94.2 | 57.1 | 42.6 | 5.8 | 94.3 | 10.4 |
| **Financial Constraints** | 122 | 68 | 20 | 42.6 | 13.2 | 79.6 | 45 | 42.2 | 20.4 | 86.8 | 20.5 |
| **Housing** | 122 | 6 | 0 | 95.1 | 0 | 100 | 0 | 95.1 | 0 | 100 | 0 |
| **Physical** | 122 | 35 | 3 | 68.9 | 0 | 96.6 | 0 | 70.6 | 3.4 | 100 | 0 |
| **Social Cohesion** | 122 | 15 | 4 | 86.1 | 6.7 | 97.2 | 25 | 88.1 | 2.8 | 93.3 | 10.5 |
| **Transportation** | 122 | 4 | 0 | 96.7 | 0 | 100 | 0 | 96.7 | 0 | 100 | 0 |

**Age < 65 (n = 292)**

| **Domain** | **N** | **Ref +** | **Pred +** | **Accuracy** | **Sensitivity** | **Specificity** | **PPV** | **NPV** | **False POS** | **False NEG** | **F1** |
| --- | --- | --- | --- | --- | --- | --- | --- | --- | --- | --- | --- |
| **Abuse** | 292 | 27 | 34 | 83.9 | 25.9 | 89.8 | 20.6 | 92.2 | 10.2 | 74.1 | 23 |
| **Alcohol** | 292 | 51 | 143 | 45.2 | 33.3 | 47.7 | 11.9 | 77.2 | 52.3 | 66.7 | 17.5 |
| **Education** | 292 | 199 | 27 | 35.6 | 9.5 | 91.4 | 70.4 | 32.1 | 8.6 | 90.5 | 16.8 |
| **Financial Constraints** | 292 | 181 | 38 | 42.1 | 13.8 | 88.3 | 65.8 | 38.6 | 11.7 | 86.2 | 22.8 |
| **Housing** | 292 | 23 | 0 | 92.1 | 0 | 100 | 0 | 92.1 | 0 | 100 | 0 |
| **Physical** | 292 | 72 | 11 | 73.6 | 4.2 | 96.4 | 27.3 | 75.4 | 3.6 | 95.8 | 7.2 |
| **Social Cohesion** | 292 | 58 | 12 | 78.1 | 5.2 | 96.2 | 25 | 80.4 | 3.8 | 94.8 | 8.6 |
| **Transportation** | 292 | 22 | 0 | 92.5 | 0 | 100 | 0 | 92.5 | 0 | 100 | 0 |

**Black (n = 94)**

| **Domain** | **N** | **Ref +** | **Pred +** | **Accuracy** | **Sensitivity** | **Specificity** | **PPV** | **NPV** | **False POS** | **False NEG** | **F1** |
| --- | --- | --- | --- | --- | --- | --- | --- | --- | --- | --- | --- |
| **Abuse** | 94 | 10 | 5 | 88.3 | 20 | 96.4 | 40 | 91 | 3.6 | 80 | 26.7 |
| **Alcohol** | 94 | 16 | 48 | 48.9 | 50 | 48.7 | 16.7 | 82.6 | 51.3 | 50 | 25 |
| **Education** | 94 | 66 | 9 | 37.2 | 12.1 | 96.4 | 88.9 | 31.8 | 3.6 | 87.9 | 21.3 |
| **Financial Constraints** | 94 | 60 | 13 | 43.6 | 16.7 | 91.2 | 76.9 | 38.3 | 8.8 | 83.3 | 27.4 |
| **Housing** | 94 | 10 | 0 | 89.4 | 0 | 100 | 0 | 89.4 | 0 | 100 | 0 |
| **Physical** | 94 | 34 | 2 | 61.7 | 0 | 96.7 | 0 | 63 | 3.3 | 100 | 0 |
| **Social Cohesion** | 94 | 16 | 5 | 77.7 | 0 | 93.6 | 0 | 82 | 6.4 | 100 | 0 |
| **Transportation** | 94 | 9 | 0 | 90.4 | 0 | 100 | 0 | 90.4 | 0 | 100 | 0 |

**Non-Black (n = 320)**

| **Domain** | **N** | **Ref +** | **Pred +** | **Accuracy** | **Sensitivity** | **Specificity** | **PPV** | **NPV** | **False POS** | **False NEG** | **F1** |
| --- | --- | --- | --- | --- | --- | --- | --- | --- | --- | --- | --- |
| **Abuse** | 320 | 24 | 44 | 83.1 | 29.2 | 87.5 | 15.9 | 93.8 | 12.5 | 70.8 | 20.6 |
| **Alcohol** | 320 | 49 | 151 | 45.6 | 26.5 | 49.1 | 8.6 | 78.7 | 50.9 | 73.5 | 13 |
| **Education** | 320 | 203 | 25 | 38.1 | 7.4 | 91.5 | 60 | 36.3 | 8.5 | 92.6 | 13.2 |
| **Financial Constraints** | 320 | 189 | 45 | 41.9 | 12.7 | 84 | 53.3 | 40 | 16 | 87.3 | 20.5 |
| **Housing** | 320 | 19 | 0 | 94.1 | 0 | 100 | 0 | 94.1 | 0 | 100 | 0 |
| **Physical** | 320 | 73 | 12 | 75.3 | 4.1 | 96.4 | 25 | 77.3 | 3.6 | 95.9 | 7.1 |
| **Social Cohesion** | 320 | 57 | 11 | 81.2 | 7 | 97.3 | 36.4 | 82.8 | 2.7 | 93 | 11.8 |
| **Transportation** | 320 | 17 | 0 | 94.7 | 0 | 100 | 0 | 94.7 | 0 | 100 | 0 |

**Female (n = 294)**

| **Domain** | **N** | **Ref +** | **Pred +** | **Accuracy** | **Sensitivity** | **Specificity** | **PPV** | **NPV** | **False POS** | **False NEG** | **F1** |
| --- | --- | --- | --- | --- | --- | --- | --- | --- | --- | --- | --- |
| **Abuse** | 294 | 29 | 30 | 84 | 20.7 | 90.9 | 20 | 91.3 | 9.1 | 79.3 | 20.3 |
| **Alcohol** | 294 | 42 | 136 | 49 | 33.3 | 51.6 | 10.3 | 82.3 | 48.4 | 66.7 | 15.7 |
| **Education** | 294 | 191 | 25 | 37.4 | 8.4 | 91.3 | 64 | 34.9 | 8.7 | 91.6 | 14.8 |
| **Financial Constraints** | 294 | 178 | 40 | 42.2 | 13.5 | 86.2 | 60 | 39.4 | 13.8 | 86.5 | 22 |
| **Housing** | 294 | 19 | 0 | 93.5 | 0 | 100 | 0 | 93.5 | 0 | 100 | 0 |
| **Physical** | 294 | 74 | 11 | 73.1 | 4.1 | 96.4 | 27.3 | 74.9 | 3.6 | 95.9 | 7.1 |
| **Social Cohesion** | 294 | 53 | 14 | 79.9 | 7.5 | 95.9 | 28.6 | 82.5 | 4.1 | 92.5 | 11.9 |
| **Transportation** | 294 | 23 | 0 | 92.2 | 0 | 100 | 0 | 92.2 | 0 | 100 | 0 |

**Male (n = 120)**

| **Domain** | **N** | **Ref +** | **Pred +** | **Accuracy** | **Sensitivity** | **Specificity** | **PPV** | **NPV** | **False POS** | **False NEG** | **F1** |
| --- | --- | --- | --- | --- | --- | --- | --- | --- | --- | --- | --- |
| **Abuse** | 120 | 5 | 19 | 85 | 60 | 86.1 | 15.8 | 98 | 13.9 | 40 | 25 |
| **Alcohol** | 120 | 23 | 63 | 40 | 30.4 | 42.3 | 11.1 | 71.9 | 57.7 | 69.6 | 16.3 |
| **Education** | 120 | 78 | 9 | 39.2 | 9 | 95.2 | 77.8 | 36 | 4.8 | 91 | 16.1 |
| **Financial Constraints** | 120 | 71 | 18 | 42.5 | 14.1 | 83.7 | 55.6 | 40.2 | 16.3 | 85.9 | 22.5 |
| **Housing** | 120 | 10 | 0 | 91.7 | 0 | 100 | 0 | 91.7 | 0 | 100 | 0 |
| **Physical** | 120 | 33 | 3 | 70 | 0 | 96.6 | 0 | 71.8 | 3.4 | 100 | 0 |
| **Social Cohesion** | 120 | 20 | 2 | 81.7 | 0 | 98 | 0 | 83.1 | 2 | 100 | 0 |
| **Transportation** | 120 | 3 | 0 | 97.5 | 0 | 100 | 0 | 97.5 | 0 | 100 | 0 |

**Supplementary Table S3. Stratified survey-to-survey agreement (research survey vs. Epic-embedded EHR survey)**

*Reference: Research Survey. Evaluated: Epic-embedded EHR Survey. Stratified by Age, Race, and Sex. Because neither instrument can be treated as a gold standard, these metrics should be read as agreement between two patient-reported sources rather than as classification performance. The Drug domain is omitted. The full-cohort cells reproduce paper Table 4.*

**Age ≥ 65 (n = 122)**

| **Domain** | **N** | **Ref +** | **Pred +** | **Accuracy** | **Sensitivity** | **Specificity** | **PPV** | **NPV** | **False POS** | **False NEG** | **F1** |
| --- | --- | --- | --- | --- | --- | --- | --- | --- | --- | --- | --- |
| **Abuse** | 122 | 3 | 7 | 91.8 | 0 | 94.1 | 0 | 97.4 | 5.9 | 100 | 0 |
| **Alcohol** | 122 | 19 | 14 | 74.6 | 5.3 | 87.4 | 7.1 | 83.3 | 12.6 | 94.7 | 6.1 |
| **Education** | 122 | 25 | 70 | 46.7 | 60 | 43.3 | 21.4 | 80.8 | 56.7 | 40 | 31.6 |
| **Financial Constraints** | 122 | 39 | 68 | 48.4 | 56.4 | 44.6 | 32.4 | 68.5 | 55.4 | 43.6 | 41.1 |
| **Housing** | 122 | 3 | 6 | 94.3 | 33.3 | 95.8 | 16.7 | 98.3 | 4.2 | 66.7 | 22.2 |
| **Physical** | 122 | 14 | 35 | 69.7 | 42.9 | 73.1 | 17.1 | 90.8 | 26.9 | 57.1 | 24.5 |
| **Social Cohesion** | 122 | 13 | 15 | 77 | 0 | 86.2 | 0 | 87.9 | 13.8 | 100 | 0 |
| **Transportation** | 122 | 13 | 4 | 87.7 | 7.7 | 97.2 | 25 | 89.8 | 2.8 | 92.3 | 11.8 |

**Age < 65 (n = 292)**

| **Domain** | **N** | **Ref +** | **Pred +** | **Accuracy** | **Sensitivity** | **Specificity** | **PPV** | **NPV** | **False POS** | **False NEG** | **F1** |
| --- | --- | --- | --- | --- | --- | --- | --- | --- | --- | --- | --- |
| **Abuse** | 292 | 15 | 27 | 86.3 | 6.7 | 90.6 | 3.7 | 94.7 | 9.4 | 93.3 | 4.8 |
| **Alcohol** | 292 | 41 | 51 | 75.3 | 24.4 | 83.7 | 19.6 | 87.1 | 16.3 | 75.6 | 21.7 |
| **Education** | 292 | 62 | 199 | 40.1 | 69.4 | 32.2 | 21.6 | 79.6 | 67.8 | 30.6 | 33 |
| **Financial Constraints** | 292 | 138 | 181 | 44.2 | 56.5 | 33.1 | 43.1 | 45.9 | 66.9 | 43.5 | 48.9 |
| **Housing** | 292 | 19 | 23 | 87 | 10.5 | 92.3 | 8.7 | 93.7 | 7.7 | 89.5 | 9.5 |
| **Physical** | 292 | 62 | 72 | 63 | 21 | 74.3 | 18.1 | 77.7 | 25.7 | 79 | 19.4 |
| **Social Cohesion** | 292 | 50 | 58 | 70.5 | 22 | 80.6 | 19 | 83.3 | 19.4 | 78 | 20.4 |
| **Transportation** | 292 | 40 | 22 | 80.1 | 5 | 92.1 | 9.1 | 85.9 | 7.9 | 95 | 6.5 |

**Black (n = 94)**

| **Domain** | **N** | **Ref +** | **Pred +** | **Accuracy** | **Sensitivity** | **Specificity** | **PPV** | **NPV** | **False POS** | **False NEG** | **F1** |
| --- | --- | --- | --- | --- | --- | --- | --- | --- | --- | --- | --- |
| **Abuse** | 94 | 5 | 10 | 84 | 0 | 88.8 | 0 | 94 | 11.2 | 100 | 0 |
| **Alcohol** | 94 | 8 | 16 | 76.6 | 12.5 | 82.6 | 6.2 | 91 | 17.4 | 87.5 | 8.3 |
| **Education** | 94 | 28 | 66 | 38.3 | 64.3 | 27.3 | 27.3 | 64.3 | 72.7 | 35.7 | 38.3 |
| **Financial Constraints** | 94 | 55 | 60 | 50 | 61.8 | 33.3 | 56.7 | 38.2 | 66.7 | 38.2 | 59.1 |
| **Housing** | 94 | 10 | 10 | 80.9 | 10 | 89.3 | 10 | 89.3 | 10.7 | 90 | 10 |
| **Physical** | 94 | 20 | 34 | 53.2 | 25 | 60.8 | 14.7 | 75 | 39.2 | 75 | 18.5 |
| **Social Cohesion** | 94 | 19 | 16 | 71.3 | 21.1 | 84 | 25 | 80.8 | 16 | 78.9 | 22.9 |
| **Transportation** | 94 | 20 | 9 | 71.3 | 5 | 89.2 | 11.1 | 77.6 | 10.8 | 95 | 6.9 |

**Non-Black (n = 320)**

| **Domain** | **N** | **Ref +** | **Pred +** | **Accuracy** | **Sensitivity** | **Specificity** | **PPV** | **NPV** | **False POS** | **False NEG** | **F1** |
| --- | --- | --- | --- | --- | --- | --- | --- | --- | --- | --- | --- |
| **Abuse** | 320 | 13 | 24 | 89.1 | 7.7 | 92.5 | 4.2 | 95.9 | 7.5 | 92.3 | 5.4 |
| **Alcohol** | 320 | 52 | 49 | 74.7 | 19.2 | 85.4 | 20.4 | 84.5 | 14.6 | 80.8 | 19.8 |
| **Education** | 320 | 59 | 203 | 43.1 | 67.8 | 37.5 | 19.7 | 83.8 | 62.5 | 32.2 | 30.5 |
| **Financial Constraints** | 320 | 122 | 189 | 44.1 | 54.1 | 37.9 | 34.9 | 57.3 | 62.1 | 45.9 | 42.4 |
| **Housing** | 320 | 12 | 19 | 91.6 | 16.7 | 94.5 | 10.5 | 96.7 | 5.5 | 83.3 | 12.9 |
| **Physical** | 320 | 56 | 73 | 68.4 | 25 | 77.7 | 19.2 | 83 | 22.3 | 75 | 21.7 |
| **Social Cohesion** | 320 | 44 | 57 | 72.8 | 15.9 | 81.9 | 12.3 | 85.9 | 18.1 | 84.1 | 13.9 |
| **Transportation** | 320 | 33 | 17 | 85.6 | 6.1 | 94.8 | 11.8 | 89.8 | 5.2 | 93.9 | 8 |

**Female (n = 294)**

| **Domain** | **N** | **Ref +** | **Pred +** | **Accuracy** | **Sensitivity** | **Specificity** | **PPV** | **NPV** | **False POS** | **False NEG** | **F1** |
| --- | --- | --- | --- | --- | --- | --- | --- | --- | --- | --- | --- |
| **Abuse** | 294 | 12 | 29 | 86.1 | 0 | 89.7 | 0 | 95.5 | 10.3 | 100 | 0 |
| **Alcohol** | 294 | 40 | 42 | 78.2 | 22.5 | 87 | 21.4 | 87.7 | 13 | 77.5 | 22 |
| **Education** | 294 | 63 | 191 | 40.8 | 63.5 | 34.6 | 20.9 | 77.7 | 65.4 | 36.5 | 31.5 |
| **Financial Constraints** | 294 | 130 | 178 | 44.2 | 55.4 | 35.4 | 40.4 | 50 | 64.6 | 44.6 | 46.8 |
| **Housing** | 294 | 15 | 19 | 89.1 | 6.7 | 93.5 | 5.3 | 94.9 | 6.5 | 93.3 | 5.9 |
| **Physical** | 294 | 59 | 74 | 63.6 | 22 | 74 | 17.6 | 79.1 | 26 | 78 | 19.5 |
| **Social Cohesion** | 294 | 43 | 53 | 71.4 | 14 | 81.3 | 11.3 | 84.6 | 18.7 | 86 | 12.5 |
| **Transportation** | 294 | 37 | 23 | 81.6 | 8.1 | 92.2 | 13 | 87.5 | 7.8 | 91.9 | 10 |

**Male (n = 120)**

| **Domain** | **N** | **Ref +** | **Pred +** | **Accuracy** | **Sensitivity** | **Specificity** | **PPV** | **NPV** | **False POS** | **False NEG** | **F1** |
| --- | --- | --- | --- | --- | --- | --- | --- | --- | --- | --- | --- |
| **Abuse** | 120 | 6 | 5 | 92.5 | 16.7 | 96.5 | 20 | 95.7 | 3.5 | 83.3 | 18.2 |
| **Alcohol** | 120 | 20 | 23 | 67.5 | 10 | 79 | 8.7 | 81.4 | 21 | 90 | 9.3 |
| **Education** | 120 | 24 | 78 | 45 | 75 | 37.5 | 23.1 | 85.7 | 62.5 | 25 | 35.3 |
| **Financial Constraints** | 120 | 47 | 71 | 48.3 | 59.6 | 41.1 | 39.4 | 61.2 | 58.9 | 40.4 | 47.5 |
| **Housing** | 120 | 7 | 10 | 89.2 | 28.6 | 92.9 | 20 | 95.5 | 7.1 | 71.4 | 23.5 |
| **Physical** | 120 | 17 | 33 | 68.3 | 35.3 | 73.8 | 18.2 | 87.4 | 26.2 | 64.7 | 24 |
| **Social Cohesion** | 120 | 20 | 20 | 75 | 25 | 85 | 25 | 85 | 15 | 75 | 25 |
| **Transportation** | 120 | 16 | 3 | 84.2 | 0 | 97.1 | 0 | 86.3 | 2.9 | 100 | 0 |

**Supplementary Table S4. Targeted chart-note error analysis of false-negative cases**

*Counts of false-negative cases (research survey = 1, SODA-derived NLP indicator = 0) assigned to each error category by chart-note review across 6 priority domains. Ten false-negative cases were randomly sampled per priority domain (n = 60 total). For each case the SODA extraction record and a keyword-based search of the linked clinical note were reviewed in parallel, and the case was assigned to one of six categories using a structured decision rule. The review was reviewed by the study team and should be read as descriptive of the kinds of discordance observed rather than as precise estimates of the proportion of all false negatives attributable to each source.*

| **Domain** | **False negatives reviewed** | **Not documented** | **Documented but not extracted** | **Ambiguous or indirect documentation** | **Documented but discordant** | **Construct mismatch** | **Unable to determine** |
| --- | --- | --- | --- | --- | --- | --- | --- |
| **Financial constraints** | 10 | 0 | 1 | 2 | 3 | 0 | 4 |
| **Abuse** | 10 | 1 | 3 | 1 | 5 | 0 | 0 |
| **Drug use** | 10 | 0 | 0 | 0 | 10 | 0 | 0 |
| **Housing** | 10 | 2 | 0 | 0 | 0 | 0 | 8 |
| **Transportation** | 10 | 2 | 0 | 0 | 3 | 0 | 5 |
| **Physical activity** | 10 | 0 | 0 | 0 | 0 | 7 | 3 |
| **TOTAL** | 60 | 5 | 4 | 3 | 21 | 7 | 20 |

**Supplementary Table S5. Item-to-binary mapping for SDoH domains across the research survey, the Epic-embedded survey, and SODA-derived indicators**

*For each of the nine SDoH domains, the table lists the survey items used and the rule applied to dichotomize each item into a binary indicator of disadvantage. The same domain definitions were applied to the research survey, the Epic-embedded survey, and the SODA-derived indicators to allow direct cross-source comparison.*

| **Domain** | **Source** | **Item / SODA category** | **Allowable responses** | **Rule for binary = 1 (disadvantage)** |
| --- | --- | --- | --- | --- |
| **Abuse** | Research survey | In the past year, have you been afraid of your partner or ex-partner? | Yes / No | Yes → 1 |
| **Abuse** | Epic-embedded survey | Within the last year, have you been (a) afraid of your partner/ex-partner; (b) humiliated or emotionally abused; (c) kicked, hit, slapped, or otherwise physically hurt; (d) raped or forced to have sexual activity by your partner/ex-partner | Yes / No / Decline | Yes to any item → 1 |
| **Abuse** | SODA / NLP | Abuse (normalized: abused / not_abused / other) | abused / not_abused / other | abused → 1 (at patient level: present if any note has abused) |
| **Alcohol use** | Research survey | How often do you have a drink containing alcohol? | Never / Monthly or less / 2-4 times/month / 2-3 times/week / 4+ times/week | 2-3 times/week or more → 1 |
| **Alcohol use** | Epic-embedded survey | During the PAST 12 MONTHS, did you drink more than a few sips of beer, wine, or any alcohol? | Yes / No | Yes → 1 |
| **Alcohol use** | SODA / NLP | Alcohol_use (normalized: yes / no / other) | yes / no / other | yes → 1 (patient-level: present if any note has yes) |
| **Drug use** | Research survey | How many times in the past year have you used an illegal drug or used a prescription medication for non-medical reasons? | Any number / No answer | Any number → 1 |
| **Drug use** | Epic-embedded survey | (Item not available in Epic SDoH survey) | N/A | N/A |
| **Drug use** | SODA / NLP | Drug_use (normalized: yes / no / other) | yes / no / other | yes → 1 (patient-level: present if any note has yes) |
| **Education** | Research survey | What is the highest level of school that you have finished? | Less than high school / High school diploma or GED / More than high school | Less than high school → 1 |
| **Education** | Epic-embedded survey | Do you have a high school degree? / Highest level of school completed | Yes / No (degree); list of education levels | Less than high school → 1 |
| **Education** | SODA / NLP | Education (normalized: based on level extracted) | (varies) | Less than high school → 1 |
| **Financial constraints** | Research survey | In the past month, how hard has it been for you to pay for the very basics like food, housing, medical care, and heating? | Not very hard / Somewhat hard / Hard / Very hard | Somewhat hard or harder → 1 |
| **Financial constraints** | Epic-embedded survey | How hard is it for you to pay for the very basics?; Food insecurity questions | Not at all / Not very hard / Somewhat hard / Hard / Very hard | Somewhat hard or harder → 1 |
| **Financial constraints** | SODA / NLP | Financial_constrain (normalized: has_financial_constraint / no_financial_constraint / other) | has_financial_constraint / no_financial_constraint / other | has_financial_constraint → 1 (patient-level: present if any note has has_financial_constraint) |
| **Housing** | Research survey | What is your housing situation today? | I have housing / I do not have housing (staying with others, hotel, shelter, outside, in car, in park) | I do not have housing → 1 |
| **Housing** | Epic-embedded survey | Housing situation as of today | I have housing / I do not have housing / Patient refused | I do not have housing → 1 |
| **Housing** | SODA / NLP | Living_Condition (normalized: stable / unstable / other) | stable / unstable / other | unstable → 1 (note: SODA has no direct housing-instability category; Living_Condition primarily captures who patient lives with) |
| **Physical activity** | Research survey | In the past 30 days, would you say that you are physically more active, less active, or about as active compared to other persons your age? | A lot less / A little less / About the same / A little more / A lot more | A lot less → 1 |
| **Physical activity** | Epic-embedded survey | On average, how many days per week do you engage in moderate to strenuous exercise?; minutes per session | Days per week (number); Minutes per session (number) | 0 days/week OR consistent with sedentary threshold → 1 |
| **Physical activity** | SODA / NLP | Physical_act (normalized: physically_active / sedentary / other) | physically_active / sedentary / other | sedentary → 1 (note: EHR field captures absolute days/week; differs from survey's relative comparison) |
| **Social cohesion** | Research survey | How often do you feel lonely or isolated from those around you? | Never / Rarely / Sometimes / Often / Always | Sometimes or more often → 1 |
| **Social cohesion** | Epic-embedded survey | In a typical week, how many times do you talk on the phone with family, friends, or neighbors? | Never / Once / Twice / Three / More than three times a week | Never → 1 (limited connectedness) |
| **Social cohesion** | SODA / NLP | Social_cohesion (normalized: high / low / other) | high / low / other | low → 1 |
| **Transportation** | Research survey | In the past 12 months, has lack of reliable transportation kept you from medical appointments, meetings, work or from getting things needed for daily living? | Yes / No | Yes → 1 |
| **Transportation** | Epic-embedded survey | In the past 12 months, has lack of transportation kept you from medical appointments or from getting medications? | Yes / No / Decline | Yes → 1 |
| **Transportation** | SODA / NLP | Transportation (normalized: has_issue / no_issue / other) | has_issue / no_issue / other | has_issue → 1 (note: Epic field is narrower [medical only] than survey scope [medical + daily living]) |
